# The capacity of diagnostic laboratories in Kenya for detecting infectious diseases

**DOI:** 10.1186/s41182-017-0049-6

**Published:** 2017-04-28

**Authors:** H-C Slotved, Kennedy K. Yatich, Shem Otoi Sam, Edwardina Otieno Ndhine

**Affiliations:** 1 Centre for Biosecurity and Biopreparedness (CBB), Statens Serum Institut, Artillerivej 5, 2300 Copenhagen, Denmark; 2 National Public Health Laboratory’s Biosafety and Biosecurity Program, Ministry of Health, Kenyatta National Hospital Grounds, Old Mbagathi Road, P.O. Box 20750-00202, Nairobi, Kenya; 3 Department of Statistics, School of Mathematics, University of Nairobi, P.O. Box 30197-00100, Nairobi, Kenya; 4 National Commission for Science, Technology and Innovation (NACOSTI), Utalii House off Uhuru Highway, P.O. Box 30623-00100, Nairobi, Kenya

**Keywords:** Kenya, Infectious diseases, Diagnostic laboratories, Capacity, Survey

## Abstract

**Background:**

The aim of this study is to present data of the diagnostic capacity of Kenyan laboratories to diagnose a number of human pathogens. The study is based on the data obtained from a biosecurity survey conducted in Kenya in 2014/2015 and data from the Statistical Abstract of Kenya for 2015. The biosecurity survey has previously been published; however, the survey also included information on laboratory capacity to handle a number of pathogens, which have not been published.

**Methods:**

Data were retrieved from the survey on 86 laboratory facilities. The data include information from relevant categories such as training laboratories, human diagnostic laboratories, veterinary diagnostic laboratories, and research laboratories.

**Results:**

The disease incidence in Kenya ranges widely from malaria and diarrhea with an incidence rate of around 10.000 per year to diseases such as cholera and yellow fever with an incidence rate of 1 per year or less for all age groups. The data showed that diseases with the highest number of diagnostic facilities were mainly malaria-, HIV-, tuberculosis-, and diarrhea-related infectious diseases.

**Conclusion:**

The study generally shows that the laboratory facilities have the capacity of detecting the infectious diseases with the highest incidence rates. Furthermore, it seems that the number of facilities able to detect a particular disease is related to the incidence rate of the disease.

## Background

Estimating and controlling the global burden of infectious diseases are main topics of many organizations [1, 2]. National disease burden data are often the information on which a country’s health system is built and focused [2]. Depending on the economic situation of a country, the health capacity of a country often varies greatly, and while many high- and upper middle-income countries are able to handle a broad spectrum of infectious diseases, this is often not the situation for the low- and low middle-income countries [3].

Kenya is a low middle-income country faced with a range of different infectious diseases, and information on the disease burden is available in Kenya [4, 5]. The annual disease data for Kenya is published by the office of Statistical Abstract of Kenya [6] and Kenya Health and Demographic Survey (KDHS) [7]. In addition, the Kenya health facility system has been described in detail by several institutions [2, 8]. Briefly, the Kenyan public health laboratory system is based on health facilities rated from level 1 (local community-based services with very limited diagnostic capacity) up to level 6 (facilities that provide national referral services with specialized health care services, including hospitals, laboratories, blood banks, and research institutions) [9]. Physically, the level system consists of 2 level 6 national referral hospitals, Moi Teaching and Referral Hospital (MTRH) and Kenyatta National Hospital (KNH), 10 level 5 regional referral hospitals, 47 level 4 county referral hospitals, and numerous level 3 and level 2 county health centers and dispensaries, which handle activities related predominantly to promotive and preventive care and various curative services. Several studies from Kenya have also described the capacity of health facilities to diagnose different diseases, including studies focusing on the capacity of health workers and laboratories to handle antimicrobial resistance [2, 3, 10].

The laboratory facilities in many low- and low middle-income countries have been found to be affected by the level of support they receive from different foreign collaborators [3]. A Kenyan study has described how the health facilities for controlling and treating diseases such as HIV/AIDS, tuberculosis, and malaria provide a high accessibility to diagnosis and treatment due to strong international support, while other diseases, both infectious diseases and chronic diseases, do not get the same attention, resulting in low accessibility to diagnosis and treatment of these diseases [3, 11].

A biosecurity survey study in Kenya has recently been published [12]. In this study, a number of laboratory facilities were visited on which occasion their general biosecurity levels were evaluated. Among the obtained information were data on the capacity of the laboratory facilities to detect various infectious diseases. The list of diseases was based on human pathogens and their biosecurity risk [12]. Using the information on the capacity of the health laboratory facilities to diagnose a number of infectious diseases [12] and the annual disease data from Kenya [6], it is the intention of this study to present data of the capacity of the Kenyan laboratory facilities and, more specifically, to possibly show how well the health systems in Kenya are focused on the infectious diseases in Kenya.

## Methods

### Disease and population data

Selected infectious disease data from Kenya were obtained from the Statistical Abstract of Kenya for 2015 [6]. All the infectious disease morbidity data are for outpatients below 5 years of age and outpatients aged 5 years and above in 2014.

**Fig. 1:**
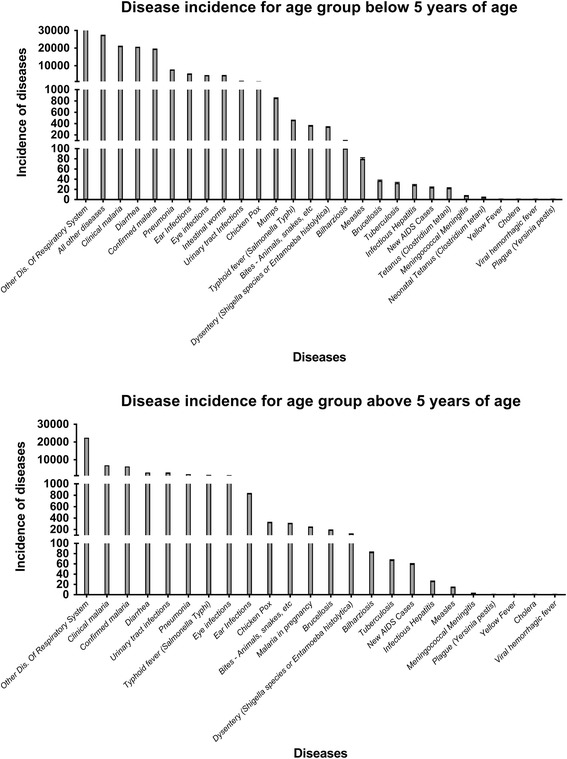
Ranked incidence rates with 95% C.I. for infectious diseases in Kenya are presented for both age <5 years and age ≥5 years of age. The data have been obtained from the Statistical Abstract of Kenya for 2015 [6]

By comparing the listed diseases in Fig. 1 with the list of diseases from the Statistical Abstract of Kenya for 2015, it can be seen which infectious disease data have been selected and used in this study.

The estimated population numbers (for 2013) used to calculate the infectious disease incidence rates for both age groups were obtained from the State of The World’s Children 2015 Country Statistical Information (UNICEF) [13]. The Kenyan population of children below 5 years of age in 2013 was 7,048,000 children. The Kenyan population of persons more than 5 years of age in 2013 was 43,648,900 people.

### Survey data

The data used in this study are from the biosecurity survey presented in the study by Ndhine et al. [12]. Briefly, the survey was based on visits and interviews in 86 selected facilities in Kenya. The relevant facility categories included 21 training and university facilities, 36 public health and hospital facilities, 11 veterinary (diagnostic) facilities, 13 foreign collaboration research facilities, 2 commercial production facilities, and 3 commercial diagnostic facilities. The laboratory facilities were situated in southern Kenya and mainly around Nairobi, Mombasa, and Kisumu.

**Fig. 2:**
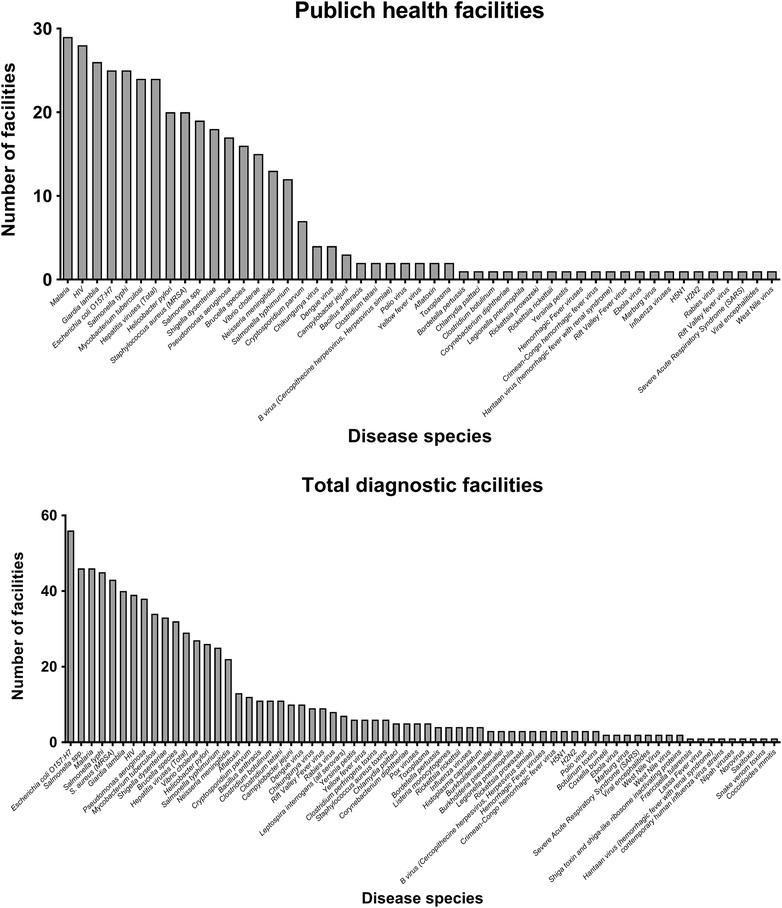
Number of laboratory facilities according to infectious diseases in Kenya presented for both the public health facilities and the total number of visited facilities

The questionnaire used in the study by Ndhine et al. [12] included questions regarding disease agents and the capacity of the facilities to handle disease agents. The nature of the work carried out in the laboratory facilities was categorized in three levels (information received as part of the responses to question 1) [12]: 1—detect specific agents (using only serological tests); 2—detect and handle (performing culture identification), but not store specimens; and 3—detect, handle, and store live specimens (identifying, isolating, and storing specimens). Figure 2 lists the disease agents, about which the laboratory facilities were asked for their capacity to handle.

When performing the questionnaire, we specifically asked for the capacity for performing a diagnostic test to detect a human disease, when talking about human health facilities. We did not ask for the quality of each specific test, as this would require an evaluation of each diagnostic test, which was not possible within this study.

All visited institutions were informed that the survey was anonymous to obtain as true and correct answers as possible.

### Data Analysis

Data were analyzed using GraphPad Prism version 5 (GraphPad Software) for descriptive statistical analysis and Excel for data handling. The adjusted incidence rates (IRs) were estimated per 100,000 person-years of observation (PYO) from reported cases per year. The number of reported disease episodes/patient was adjusted for the total reporting rates. For further details, see the Statistical Abstract of Kenya for 2015 [7].

## Results

The listed disease incidence in Kenya shows a very wide range from malaria and diarrhea with an incidence rate of around 10,000 or more to diseases such as cholera and yellow fever with an incidence rate of around 1 or less for all age groups (Fig. 1, Tables 1 and 2). In general, the incidence rates are similar for both age groups, although some diseases show an age-dependent difference, e.g., the incidence rate for mumps in children <5 years of age was 848.6 (95% C.I. 841.9–855.4) while it was found to be 0 for the age group ≥5 years in 2014.

**Table 1:** Incidence of disease age <5 years

	List of diseases 2014 Kenya (age <5 years)	Adjusted incidence rate^a^ (95% C.I.)	Total facilities 86 facilities *N* (%)	Public health facilities 36 facilities *N* (%)
1	Other dis. of respiratory system	87,866.3 (87,842.2–87,890.4)	Partial	Partial
2	Malaria (clinical)	21,073.6 (21,043.5–21,103.7)	46 (53%)	29 (81%)
3	Diarrhea	20,548.0 (20,518.1–20,577.8)	Partial	Partial
4	Pneumonia	7633.5 (7613.9–7653.1)	No information	No information
5	Chicken pox	1164.8 (1156.9–1172.8)	Partial	No information
6	Mumps	848.6 (841.9–855.4)	No information	No information
7	Typhoid fever (*Salmonella* Typhi)	456.9 (451.9–461.9)	45 (52%)	25 (69%)
8	Dysentery (*Shigella* species or *Entamoeba histolytica*)	341.7 (337.4–346.0)	Partial *Shigella dysenteriae* (33%)	Partial *Shigella dysenteriae* (18%)
9	Bilharziosis	101.3 (99.0–103.7)	No information	No information
10	Brucellosis	37.0 (35.6–38.4)	32 (37%)	16 (44%)
11	Tuberculosis	32.6 (31.3–33.9)	34 (40%)	24 (67%)
12	Infectious hepatitis	28.5 (27.2–29.7)	29 (34%)	24 (67%)
13	New AIDS cases	23.7 (22.6–24.8)	39 (45%)	28 (78%)
14	Tetanus (*Clostridium tetani*)	22.3 (21.3–23.5)	11 (13%)	2 (6%)
15	Meningococcal meningitis	7.3 (6.7–8.0)	22 (26%)	13 (36%)
16	Yellow fever	0.9 (0.7–1.1)	6 (7%)	2 (6%)
17	Viral hemorrhagic fever	0.1 (0.06–0.2)	Partial	Partial
18	Plague (*Yersinia pestis*)	0.05 (0.02–0.14)	6 (7%)	1 (3%)

^a^Adjusted data (based on reporting rates) according to the information provided by KNBS [6]

**Table 2:** Incidence of disease age ≥5 years

	List of diseases 2014 Kenya (age ≥5 years)	Adjusted incidence rate^a^ (95% C.I.)	Total facilities 86 facilities *N* (%)	Public health facilities 36 facilities *N* (%)
1	Other dis. of respiratory system	22,421.4 (22,109.0–22,433.8)	Partial	Partial
2	Malaria (clinical)	6865.8 (6858.3–6873.3)	46 (53%)	29 (81%)
3	Typhoid fever (*Salmonella* Typhi)	1496.5 (1492.9–1500.1)	45 (52%)	25 (69%)
4	Chicken pox	325.4 (323.7–327.1)	Partial	No information
5	Brucellosis	192.2 (190.9–193.5)	32 (37%)	16 (44%)
6	Dysentery (*Shigella* species or *Entamoeba histolytica*)	123.7 (122.7–124.7)	Partial *Shigella dysenteriae* (33%)	Partial *Shigella dysenteriae* (18%)
7	Bilharziosis	82.6 (81.7–83.4)	No information	No information
8	Tuberculosis	67.4 (66.7–68.2)	34 (40%)	24 (67%)
9	New AIDS cases	59.9 (59.2–60.7)	39 (45%)	28 (78%)
10	Infectious hepatitis	26.2 (25.8–26.7)	29 (34%)	24 (67%)
11	Measles	14.7 (14.3–15.0)	No information	No information
12	Meningococcal meningitis	2.9 (2.7–3.0)	22 (26%)	13 (36%)
13	Plague (*Yersinia pestis*)	1.0 (0.9–1.1)	6 (7%)	1 (3%)
14	Yellow Fever	0.5 (0.48–0.62)	6 (7%)	2 (6%)
15	Cholera	0.3 (0.29–0.4)	27 (31%)	15 (42%)
16	Viral hemorrhagic fever	0.25 (0.21–0.3)	Partial	Partial

^a^Adjusted data (based on reporting rates) according to the information provided by KNBS [6]

In Fig. 2 and Table 3, the number of laboratory facilities is listed according to the diseases that they stated they were able to detect and diagnose. In general, the diseases with the highest number of laboratory facilities were malaria, HIV, tuberculosis, and diarrheal infectious diseases.

**Table 3:** The incidence of diseases listed according to the highest number of facilities with diagnostic capacity

	Adjusted incidence rate^a^ (95% C.I.)	Total facilities; number of facilities (*N* = 86 facilities)		Adjusted incidence rate^a^ (95% C.I.)	Public health facilities; number of facilities (*N* = 36 facilities)
*Escherichia coli*	Unknown	56 (65%)	Malaria (clinical)	<5 years 21,073.6 (21,043.5–21,103.7) ≥5 years 6865.8 (6858.3–6873.3)	29 (81%)
*Salmonella* spp.	Unknown	46 (53%)	New AIDS cases	<5 years 23.7 (22.6–24.8) ≥5 years 59.9 (59.2–60.7)	28 (78%)
Malaria (clinical)	<5 years 21,073.6 (21,043.5–21,103.7) ≥5 years 6865.8 (6858.3–6873.3)	46 (53%)	*Giardia lamblia*	Unknown	26 (72%)
*Salmonella* Typhi	<5 years 456.9 (451.9–461.9) ≥5 years 1496.5 (1492.9–1500.1)	45 (52%)	*Escherichia coli*	Unknown	25 (69%)
*Staphylococcus aureus*	Unknown	43 (50%)	*Salmonella* Typhi	<5 years 456.9 (451.9–461.9) ≥5 years 1496.5 (1492.9–1500.1)	25 (69%)
*Giardia lamblia*	Unknown	40 (47%)	*Mycobacterium tuberculosis*	<5 years 32.6 (31.3–33.9) ≥5 years 67.4 (66.7–68.2)	24 (67%)
New AIDS cases	<5 years 23.7 (22.6–24.8) ≥5 years 59.9 (59.2–60.7)	39 (45%)	Hepatitis	<5 years 28.5 (27.2–29.7) ≥5 years 26.2 (25.8–26.7)	24 (67%)
*Pseudomonas aeruginosa*	Unknown	38 (44%)	*Helicobacter pylori*	Unknown	20 (56%)
*Mycobacterium tuberculosis*	<5 years 32.6 (31.3–33.9) ≥5 years 67.4 (66.7–68.2)	34 (40%)	*Staphylococcus aureus*	Unknown	20 (56%)
*Shigella dysenteriae*	Partial <5 years 28.5 (27.2–29.7) Partial ≥5 years 26.2 (25.8–26.7)	33 (38%)	*Salmonella* species	Unknown	19 (53%)
*Brucella* species	<5 years 37.0 (35.6–38.4) ≥5 years 192.2 (190.9–193.5)	32 (37%)	*Shigella dysenteriae*	Partial <5 years 28.5 (27.2–29.7) Partial ≥5 years 26.2 (25.8–26.7)	18 (50%)
Hepatitis	<5 years 28.5 (27.2–29.7) ≥5 years 26.2 (25.8–26.7)	29 (34%)	*Pseudomonas aeruginosa*	Unknown	17 (47%)
*Vibrio cholerae*	<5 years –––––––––––– ≥5 years 0.3 (0.29–0.4)	27 (31%)	*Brucella* species	<5 years 37.0 (35.6–38.4) ≥5 years 192.2 (190.9–193.5)	16 (44%)
*Helicobacter pylori*	Unknown	26 (30%)	*Vibrio cholerae*	<5 years ––––––––– ≥5 years 0.3 (0.29–0.4)	15 (42%)
*Salmonella* Typhimurium	Unknown	25 (29%)	*Neisseria meningitidis*	<5 years 7.3 (6.7–8.0) ≥5 years 2.9 (2.7–3.0)	13 (36%)
*Neisseria meningitidis*	<5 years 7.3 (6.7–8.0) ≥5 years 2.9 (2.7–3.0)	22 (26%)	*Salmonella* Typhimurium	Unknown	12 (33%)

^a^Adjusted data (based on reporting rates) according to the information provided by KNBS [6]

Tables 1 and 2 show the number of facilities capable of detecting the infectious diseases listed, e.g., in the Statistical Abstract of Kenya. The diseases are listed according to the incidence rate, showing the highest incidence rate at the top.

For the infectious diseases with the highest incidence rates for patients aged <5 years and from which facility information was available, it was found that more than one third of the health facilities was able to detect the diseases, except for tetanus (two health facilities), plague (one health facility), and yellow fever (two health facilities). The disease incidence rates (<5 years) for tetanus (22.3 (95% C.I. 21.3–23.5)), plague (0.05 (95% C.I. 0.02–0.14)) and yellow fever (0.9 (95% C.I. 0.7–1.14)) were generally low.

For the infectious diseases with the highest incidence rates for patients aged ≥5 years and from which facility information was available, it was found that more than one third of the health facilities was able to detect the diseases, except for plague (one health facility) and yellow fever (two health facilities). The incidence rates for plague (1.0 (95% C.I. 0.9–1.1)) and yellow fever (0.55 (95% C.I. 0.48–0.62)) were very low.

## Discussion

The specimen referral system in Kenya is described in the Kenya Health Sector Referral Strategy [9]; in general the specimens are sent from one level up to the next level, where the required analysis is being performed. Laboratories (Levels) can bypass the next level in line to reach the level, which has the required analysis, based on the minimum standard requirement known for each level [9]. More specific details and illustrations on the Kenyan health systems structure can be found in the Kenya Health Sector Referral Strategy (2014–2018) [9]. Several quality control studies regarding the performance of Ministry of Health (MoH) laboratories have been performed [10, 14]. The quality of the microbiology performed in county hospital laboratories (levels 4 and 5) has been described as low [10, 14]. As presented in the Referral Networks Strengthening Laboratory Health Systems in Kenya [15], many organizations are involved in developing and improving the Kenyan health system; some of the major stakeholders are WHO, CDC, UNICEF, and the World Bank. Based on Integrated Disease Surveillance and Response guidelines (IDSR guidelines, WHO), these stakeholders do try to improve the Kenyan health sector. The IDSR guidelines [16] describe how to handle the disease surveillance and response, and in particular, CDC and WHO are involved in these guidelines [16].

In the study by Prince and Otieno [3], it was found that in particular, three infectious diseases have an international focus with huge international support available. These diseases are HIV/AIDS, tuberculosis, and malaria. Looking at the incidences for the three diseases, they all show relatively high incidence rates (Tables 1 and 2). Compared to other diseases, only malaria was found to be the top incidence disease, while other diseases showed higher incidence rates than tuberculosis and new AIDS cases. Compared to the number of laboratory facilities capable of detecting diseases, it was found that all three diseases are detected at a relatively higher number of facilities (Table 3). More than two thirds of the health facilities stated that they were able to detect HIV/AIDS, tuberculosis, and malaria, even though they were not the top three diseases regarding incidence rate. It can furthermore be speculated that the incidence rates for the three diseases are high because the facilities are able to detect them, whereas the incidence rates of several other diseases might be lower since facilities are not able to detect these diseases.

The questionnaire [12] from which the data for this study were obtained, generally only focused on specific infectious diseases but not on the antibiotic susceptibility of bacteria causing infectious diseases. However, resistance is a major problem in Kenya, and the extent of this problem is not known in details, although some of the resistance problems are due to the lack of diagnostic equipment such as culturing facilities and availability of susceptibility tests [17, 18]. Among the species with known resistance problems are *Salmonella* species, *Escherichia coli*, and *Staphylococcus aureus* [17].

The Kenyan health facilities also have to be prepared for pathogens from other parts of the world, as shown by Wong et al. [19]. They showed that a transmission of multidrug resistance *Salmonella* Typhi strains had appeared from India to Kenya. One of the participating health institutions from the questionnaire [12] also raised this concern (data not shown). As shown in Table 3, a high proportion of the laboratory facilities were able to detect *S.* Typhi; however, data on the capacity to measure the antibiotic susceptibility of these strains could not be obtained.

The limitation of this study is that the laboratory data are based on the interest in biosecurity [12] rather than general disease. It is furthermore a limitation of this study that only few private laboratory facilities for human health were visited and evaluated for their diagnostic capacities. There is a large number of private clinical laboratories in Kenya [20]. In general, a large part of the private laboratories are well equipped, have high-quality laboratory facilities, and are able to perform many different kinds of microbiological tests, including culturing. Some of the laboratories refer the specimen to level 6 laboratories or other private laboratories in and outside Kenya, depending on what kind of collaborations they have. Several of the laboratories have branches in other African countries, where they have a centrally placed high-quality reference laboratory in, for example, South Africa [21].

## Conclusions

In conclusion, the survey data on Kenyan laboratory facilities in general show that they have the capacity of detecting the infectious diseases with the highest incidence rate. It furthermore seems that the number of facilities able to detect a particular disease is related to the disease incidence rate. Within the limitation of the questionnaire data, this study finds that there is a relatively good correlation between the number of facilities able to detect a specific infectious disease and how high the particular infectious disease rate burden is for Kenya.

## Abbreviations

IDSR guidelines, WHO: Integrated Disease Surveillance and Response guidelines
IRs: Adjusted incidence rates
KDHS: Kenya Health and Demographic Survey
KNH: Kenyatta National Hospital
MoH: Ministry of Health
MTRH: Moi Teaching and Referral Hospital
PYO: Per 100,000 person-years of observation
